# Selenium Attenuates Ethanol-induced Hepatocellular Injury by Regulating Ferroptosis and Apoptosis

**DOI:** 10.5152/tjg.2024.24159

**Published:** 2024-10-01

**Authors:** Feng Chen, Qianhui Li, Xiaomin Xu, Fei Wang

**Affiliations:** Division of Gastroenterology, Seventh Affiliated Hospital of Sun Yat-sen University, Shenzhen, China

**Keywords:** Selenomethionine, ebselen, hepatocytes, ferroptosis, alcoholic liver disease

## Abstract

**Background/Aims:**

Ferroptosis is a newly identified type of cell death which is strongly linked to the development of several diseases. Whereas, the role of ferroptosis in the improvement of ethanol-induced hepatocytes injury by selenium has not been confirmed.

**Materials and Methods:**

In this study, an in vitro cell damage model was established using half inhibition concentration of ethanol to induce NCTC clone 1469. Cell activity, lipid peroxidation, apoptosis and the expression of markers related to ferroptosis pathway was determined. A mouse model of alcoholic liver disease (ALD) was constructed and the effectiveness of selenium and ferrostatin-1 in treating ALD in vivo was assessed by serum liver function tests, tissue staining and immunohistochemistry for ferroptosis related proteins.

**Results:**

Pretreatment with selenomethionine and ebselen significantly improved ethanol-induced reduction in hepatocyte viability, elevated GSH levels and SOD enzyme activity, reduced MDA and iron content, while improving ethanol-induced changes in apoptosis levels and ferroptosis markers GPX4, SLC7A11, and ACSL4, with the effect of Selenomethionine being more significant. In vivo results also indicated that intervention with selenium or ferroptosis inhibitors significantly improved ethanol-induced liver tissue damage, significantly reduced serum ALT and AST levels, upregulated GPX4 and SLC7A11, but reduced ACSL4 protein levels in liver tissue.

**Conclusion:**

The process of ethanol damage to hepatocytes is regulated by the ferroptosis pathway. Selenium may exert a beneficial role in ethanol-induced hepatocyte injury by antagonizing oxidative stress and regulating apoptosis and ferroptosis pathways.

**Main Points**

- The process of ethanol damage to hepatocytes is regulated by the ferroptosis pathway.
- Selenium compounds or ferroptosis inhibitors can effectively alleviate ALD.
- Selenium may play a therapeutic role in ethanol-induced hepatocyte injury by antagonizing oxidative stress and regulating apoptosis and ferroptosis pathways.

## Introduction

Cell death occurs throughout the normal functioning of the organism as well as in physiopathological processes, and hepatocyte death is an important pathogenic factor in advancing various liver diseases. To date, several types of cell death, such as autophagy, apoptosis, pyroptosis, and necrosis, have been shown to be associated with the cytotoxic effects of alcohol metabolism.^1^ In addition, lipid peroxidation and iron overload during alcohol metabolism have been well documented, suggesting another form of cell death in alcoholic liver disease (ALD).^2-3^ Ferroptosis is a newly discovered form of regulatory cell death driven by lipid peroxidation and iron accumulation. Excess iron deposition, generation of reactive oxygen species (ROS), aggregation of lipid peroxides, and the failure of glutathione peroxidase 4 (GPX4) activity are all key factors in inducing the onset of ferroptosis.^4^ Iron overload in the liver is a major causative factor in various types of liver injury diseases and is also associated with liver fibrosis. Several investigations indicated that ferroptosis is implicated in the pathological processes of various hepatic diseases, for instance ALD, viral hepatitis, non-alcoholic steatohepatitis, drug-induced liver injury, and hemochromatosis.^3^ Moreover, hepatocyte ferroptosis may be an important factor in inducing disease progression and exacerbating liver pathological manifestations. Therefore, artificial intervention to induce or inhibit ferroptosis by small molecule compounds would open new opportunities for the treatment of the disease.

Selenium has broad biological functions, mainly as selenium-containing proteins and amino acids that exist in the body. Various human diseases are closely related to selenium deficiency, and extensive research has been conducted due to its beneficial preventive and adjuvant therapeutic effects on malignant tumors. Selenium is a known essential component for the synthesis of glutathione peroxidase, with the GPX4 protein containing the active site selenocysteine. Studies suggest that selenium supplementation improves the body’s resistance to ferroptosis, and selenium deficiency affects the body’s susceptibility to ferroptosis to some extent by regulating the abundance and activity of the GPX4 protein,^5-6^ and selenium-containing compounds are currently being investigated as one of the targeted drugs for clinical therapeutic strategies against ferroptosis. In ALD patients, the synthesis of relevant selenoproteins in the liver is inhibited by ethanol-induced oxidative damage, leading to an impaired antioxidant defense system, which exacerbates inflammatory responses and lipid metabolism disorders, and worsens ALD symptoms. Selenium supplementation has an antioxidant effect in improving ALD.^7^ However, reports on the use of selenium compounds to improve ALD by targeting ferroptosis are still limited. Therefore, in this study, we focus on analyzing the role of 2 selenium compounds, selenomethionine (Se-M) and ebselen (Eb), in ethanol-induced hepatocyte injuryin vitroand in vivo, while exploring the forms of cell death involved.

## Materials and Methods

### Reagents and Materials

All cell culture consumables and reagents were bought from Corning Incorporated (Corning, NY, USA) or Gibco (Carlsbad, Calif, USA). selenomethionine and the Iron assay kit were purchased from Sigma-Aldrich (St. Louis, Mo, USA). Ebselen and ferrostatin-1 (fer-1) were obtained from Cayman (Mich, USA). CellTiter-glo^®^ luminescent cell viability assay was from Promega Corporation (Beijing, China). All antibodies and the ALT/AST ELISA kit were purchased from Abcam (Cambridge, UK) and ABclonal Technology (Wuhan, China). The caspase 3/7 activity apoptosis assay kit was obtained from AAT Bioquest (Sunnyvale, Calif, USA). Kits used for superoxide dismutase (SOD), reduced glutathione (GSH), malondialdehyde (MDA), and apoptosis detection were purchased from Beyotime Technology (Shanghai, China). The quantitative real-time polymerase chain reaction (qRT-PCR)-related reagents were obtained from TransGen Biotech (Beijing, China), unless otherwise specified.

### Cell Culture and Treatment

Mouse normal hepatocytes (NCTC clone 1469) were purchased from the China Center for Type Culture Collection. The normal cell culture medium was Dulbecco‘s modified Eagle medium (DMEM) plus 10% horse serum. The culture medium that contains 0, 10, 25, 50, 100, 200, 400, 800, and 1600 mM of ethanol was used to treat the cells, respectively. Cell viability was measured after 4 h of incubation,^8-9^ and the ethanol concentration used to establish the cellular oxidative damage model was determined according to the half inhibition concentration (IC50). In addition, selenomethionine or ebselen containing 0, 0.01, 0.1, 0.25, 0.5, 1.0, 2.0, 5.0, and 10.0 μM were added to the cell culture medium, and the appropriate concentrations for subsequent experiments were selected based on the results of cell viability measurements after 24-h incubation.

### Cell Viability Measurements

Cell viability changes were performed using the CellTiter-Glo® luminescence assay kit. The buffer solution was mixed with the substrate bottle, and 100 μL of CellTiter-Glo mixture reagent was added to each well of the treated 96-well plate cellsand then placed the well plate into a fixed rail shaker and mixed to induce cell lysis. To stabilize luminescent signal, the plate was incubated at room temperature for 10 min. Finally, the luminescence at 560 nm was measured in a microplate reader with cell-free culture medium serving as a blank control.

### Lipid Peroxidation and Iron Content Assay

MDA content, GSH content, SOD enzyme activity level, and iron content in the cell lysates of each treatment group were determined using commercial kits following the manufacturer‘’s instructions to evaluate the changes in endogenous lipid peroxidation levels and iron content.

### Apoptosis Assay

The cultured cells’ apoptosis level was detected using the TransDetect^®^ Annexin V-FITC/PI Apoptosis Assay Kit. The procedure was performed according to the instructions, and the percentage of apoptosis was analyzed by flow cytometry. Changes in caspase-3/7 activity in cell lysates of each treatment group were measured using the Cell Meter Caspase 3/7 Activity Apoptosis Assay Kit following the manufacturer‘’s instructions, and the results were read in a microplate reader at 520 nm, with a fold change in expression compared to the control.

### qRT-PCR Analysis

Total RNA was extracted from the cells using AG RNAex Pro RNA reagent, and RNA concentration was determined by Nano Drop 2000 spectrophotometer. PCR amplification was performed using Transcript^®^ Probe One-Step qRT-PCR SuperMix Kit and CFX-96 real-time PCR system (Bio-Rad, USA) to detect ferroptosis pathway-related genes* GPX4*, Solute Carrier Family 7 Member 11 (*SLC7A11*), and Acyl-CoA Synthetase Long Chain Family Member 4 (*ACSL4*), *GADPH* was used as an internal reference for normalization, and 2^−ΔΔCt^ method was used to calculate the relative expression levels of the genes. Each experiment was replicated independently at least 3 times. Primers were synthesized by GuangZhou TianYi HuiYuan Gene Technology Co., Ltd., and the sequences are shown below: *GPX4* (forward: 5’-CCTCCCCAGTACTGCAACAG-3’; reverse: 5’-GGCTGAGAATTCGTGCATGG-3’), *SLC7A11* (forward: 5’-TGGGTGGAACTGCTCGTAAT-3‘; reverse: 5’-AGGATGTAGCGTCCAAATGC-3’), *ACSL4* (forward: 5’-CCACACTTATGGCCGCTGTT-3’; reverse: 5’-GGGCGTCATAGCCTTTCTTG-3’), *GAPDH* (forward: 5’-TGACCTCAACTACATGGTCTACA-3’; reverse: 5’-CTTCCCATTCTCGGCCTTG-3’).

### Immunofluorescence Analysis

After the specified treatments, the cells were immobilized with 4.0% paraformaldehyde solution, infiltrated with 0.3% Triton X-100, and blocked with 1.0% bovine serum albumin. Diluted SLC7A11 (1:500), GPX4 (1:100), and ACSL4 (1:200) antibody solutions were added according to the antibody instructions. The Cy3-labeled secondary antibodies were incubated in the dark at room temperature, and 4’,6-diamidino-2-phenylindole (DAPI) was used for nuclear staining. Immunofluorescence imaging was performed by inverted fluorescence microscopy (DMi8; Leica, Wetzlar, Germany).

### Animal Experiments

All animal experiments were conducted in accordance with the ARRIVE guidelines and the Guide for the Care and Use of Laboratory Animals (8th edition). The experiments were approved by the Animal Ethics Committee of Sun Yat-sen University (approval number: SYSU-IACUC-2021-000349; date: May 13, 2021). Eight-week-old male normal C57BL/6 mice were bought from Shanghai SLAC Laboratory Animal Co., Ltd. The diets were derived from Trophic Animal Feed High-tech Co., Ltd (Jiangsu, China). After 5 days of adaptive feeding, the mice were randomized into control, ALD, ALD+Se, and ALD+fer-1 treatment groups, with 6 mice in each group. According to the chronic alcoholic liver injury induction protocol announced by the National Institute on Alcohol Abuse and Alcoholism (NIAAA),^10^ C57BL/6 mice were freely fed with Lieber-DeCarli ethanol liquid diet (5% v/v) for 10 days, with a 20% ethanol binge (5 g/kg, body weight) on the last day. Mice in the Se and fer-1 treatment groups were intraperitoneally injected with selenomethionine (25 μg/kg body weight) dissolved in H_2_O and ferrostatin-1 (2.5 μmol/kg body weight) dissolved in 1% dimethyl sulfoxide solution, respectively, for 10 consecutive days starting 1 day before the alcohol liquid diet feeding. Mice in the control group were injected with equal amounts of phosphate buffer solution. Liver tissue and serum were collected at the endpoint of the experiment, i.e., 9 h after binge drinking.

### Serum and Liver Tissue Analysis

Whole blood was taken and centrifuged at 1000 × g for 10 min at 4 °C, and the serum levels of ALT and AST were detected according to the manufacturer’s instructions. Liver tissues were embedded in paraffin and morphological changes were observed by H&E and Sirius red staining using polarized light microscopy (Leica, Wetzlar, Germany). The expression of specific antigens of SLC7A11, GPX4, and ACSL4 was detected by immunohistochemical.

### Statistical Analysis

SPSS version 26.0 (IBM SPSS Corp.; Armonk, NY, USA) statistical software was applied for data analysis, and 1-way ANOVA was first used for comparison between multiple groups. If the differences between groups were statistically significant, further 2-way comparisons were performed using the *LSD-t* test, with *P *< .05 as the difference being statistically significant.

## Results

### Selenium Attenuated Ethanol-induced Hepatocytes Damage in Vitro

**Figure 1.**
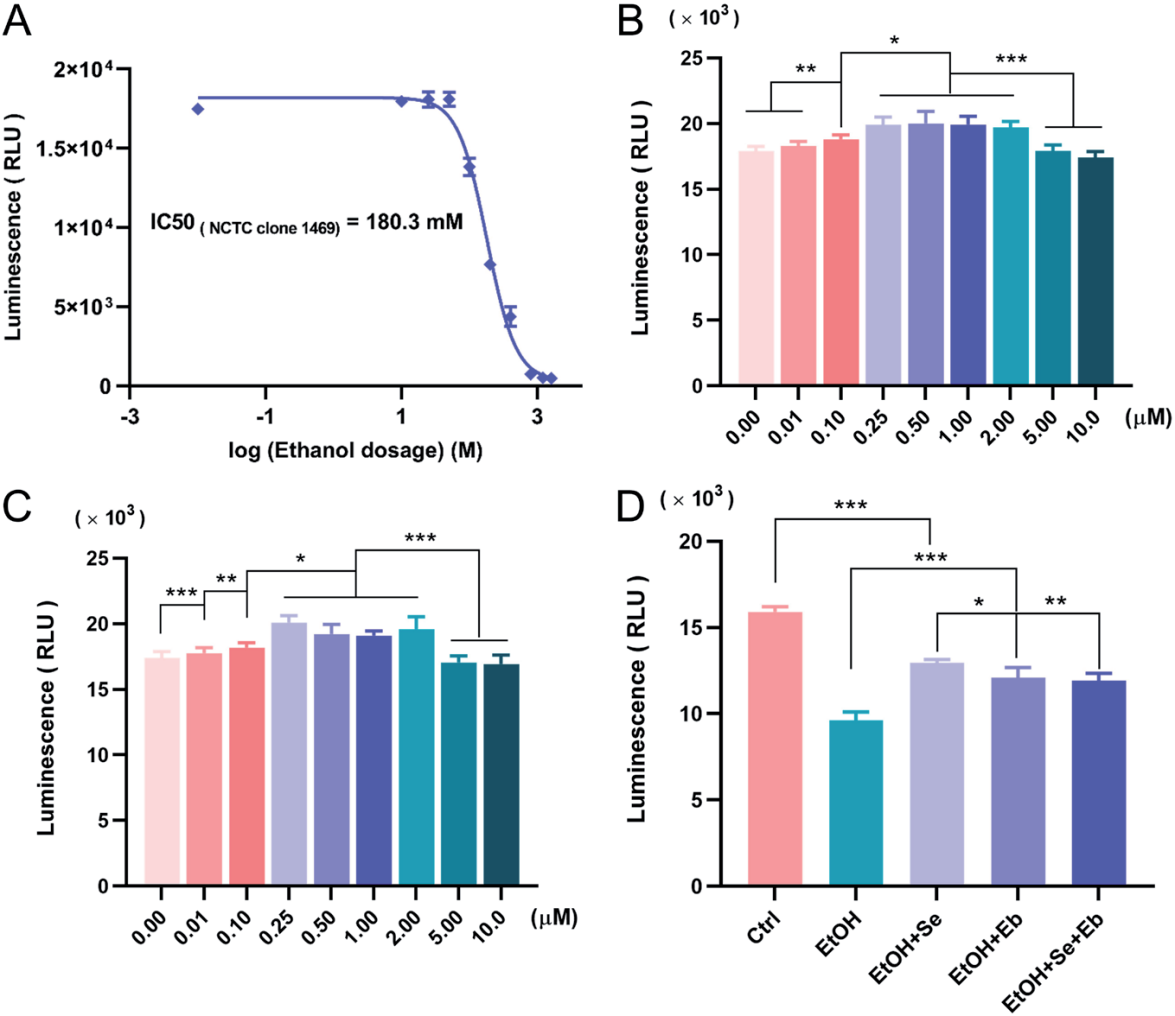
Effects of Selenomethionine (Se) and Ebselen (Eb) on ethanol-induced hepatocyte damage *in vitro*. (A) The half maximal inhibitory concentration (IC50) of ethanol to NCTC clone 1469. Cells were pre-treated with indicated concentrations of ethanol for 4 h and the IC50 was determined. (B–C) Effects of Se (B) and Eb (C) to NCTC clone 1469. Cells were pre-treated with indicated concentrations of selenium compounds for 24 h and cell activity changes were detected. (D) Changes in NCTC clone 1469 viability after ethanol intoxication in the presence of Se and/or Eb treatments. Data from each group were expressed as mean ± SD (*n *= 4). * means* P *< .05, ** means* P *< .01 and *** means* P *< .001 between indicated groups.

Cell cultures containing different concentrations of ethanol were added to NCTC clone 1469, and the results showed that the IC50 value of ethanol-induced damage in NCTC clone 1469 was 180.3 mM (Figure 1A). Experiments on the effects of different selenium compounds on cell proliferation showed that the cell viability of the selenomethionine and Eb groups, ranging from 0.25 to 2.0 μM, was significantly higher than that of the control, 5.0 μM, and 10.0 μM groups, with statistically significant differences (all *P *< .001) (Figure 1B and C). Therefore, we selected a selenium compound concentration of 2.0 μM as the final dose for subsequent experiments. It was further observed that the addition of 2.0 μM Se and/or Eb to the cell culture medium 24 h prior to ethanol exposure was effective in increasing cell viability compared with the ethanol group (all *P *< .001), with Se pretreatment showing the best recovery effect (Figure 1D).

### Selenium Alleviated Ethanol-induced Lipid Peroxidation and Iron Accumulation in Hepatocytes

**Figure 2.**
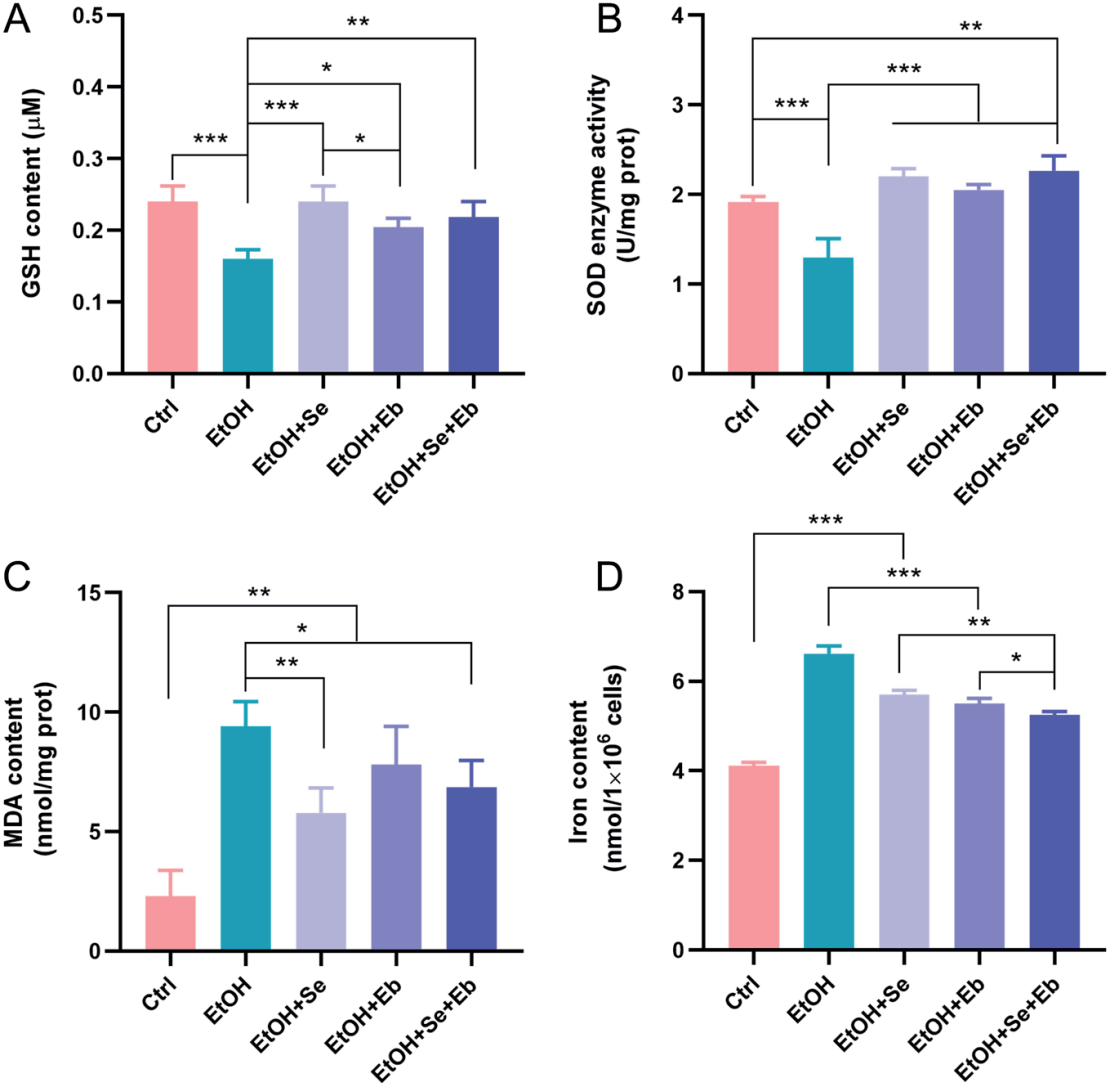
Selenomethionine (Se) and/or ebselen (Eb) treatment alleviate ethanol-induced lipid peroxidation and iron accumulation in hepatocytes. The GSH content (A), SOD enzyme activity (B), MDA (C), and iron content (D) in the NCTC clone 1469 after treatments with or without ethanol, Se, or Eb was detected by corresponding commercial kits. Data from each group were expressed as mean ± SD (n = 3). * means* P *< .05, ** means* P *< .01, and *** means* P *< .001 between indicated groups.

To assess the changes in antioxidant index levels and iron content of selenium compounds in ethanol-induced hepatocyte injury, we examined MDA, GSH, iron content, and SOD enzyme activity. Compared to the control group, GSH and SOD enzyme activity were significantly decreased in the ethanol-treated group, while MDA and iron contents were significantly increased, with significant differences (all *P *< .001). GSH content (*P *< .001, *P *= .015, and *P *= 0.003, respectively) and SOD enzyme activity (all *P *< .001) were significantly higher, and iron content (all *P *< .001) was lower in the Se, Eb, and Se+Eb intervention groups compared to the ethanol-treated group. However, only the Se (*P *= .004) and Se+Eb (*P *= .026) intervention groups showed a significant decrease in MDA content. The changes in antioxidant indexes were more pronounced in the Se intervention group. Although there were more significant changes compared to the ethanol-treated group, but the intervention with selenium compound groups still had significantly higher levels of MDA (all *P *< .01) and iron content (all *P *< .001) than those of the control group (Figure 2).

### Effect of Selenium on Ethanol-induced Apoptosis Level in Hepatocytes

**Figure 3.**
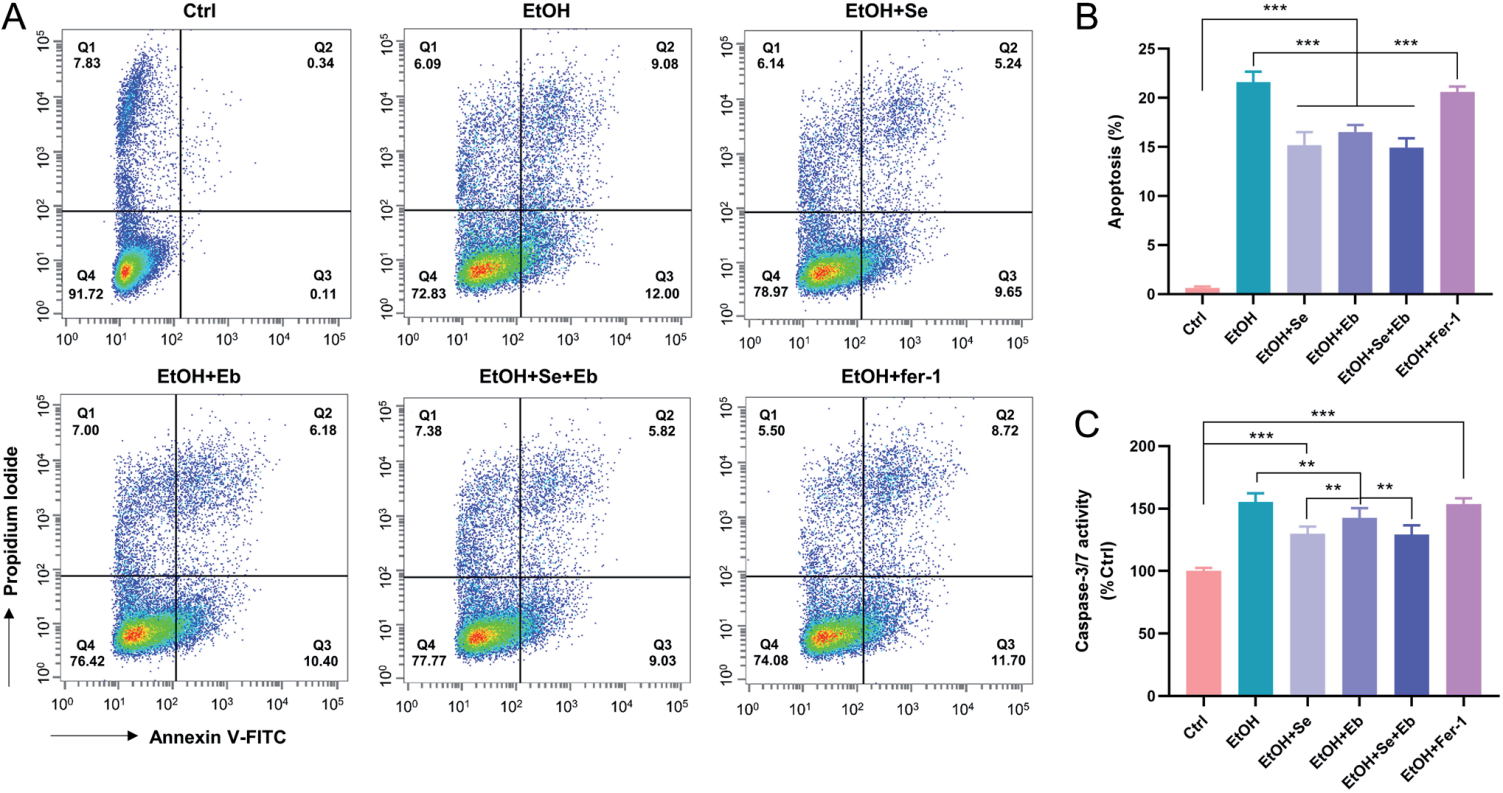
Effect of selenium compounds and ferrostatin-1 on the level of ethanol-induced apoptosis in hepatocytes. (A-C) The changes in apoptosis levels (A, B) and caspase-3/7 activity (C) of NCTC clone 1469 after treatments with or without ethanol, selenomethionine (Se), ebselen (Eb), ferrostatin-1 (fer-1) were detected. The total proportion of apoptotic cells was Q2+Q3. Data from each group were expressed as mean ± SD (n= 4). * means* P *< .05, ** means* P *< .01 and *** means* P *< .001 between indicated groups.

The changes in apoptosis levels among the different treatment groups were detected. The results (Figure 3) showed that ethanol treatment induced more than 20% cell apoptosis compared with the control group. The addition of Se and/or Eb pretreatment significantly diminished the percentage of apoptosis compared with the ethanol group (all *P *< .001), while no significant changes were observed in the group pretreated with fer-1 (1.0 μM) (*P *= .183). This result was further supported by changes in caspase-3/7 activity and suggested that selenium compounds may partially alleviate ethanol-induced hepatocyte injury by modulating the expression of apoptotic molecules.

### Selenium Modulated Ethanol-induced Hepatocyte Injury Through Ferroptosis Pathway

**Figure 4.**
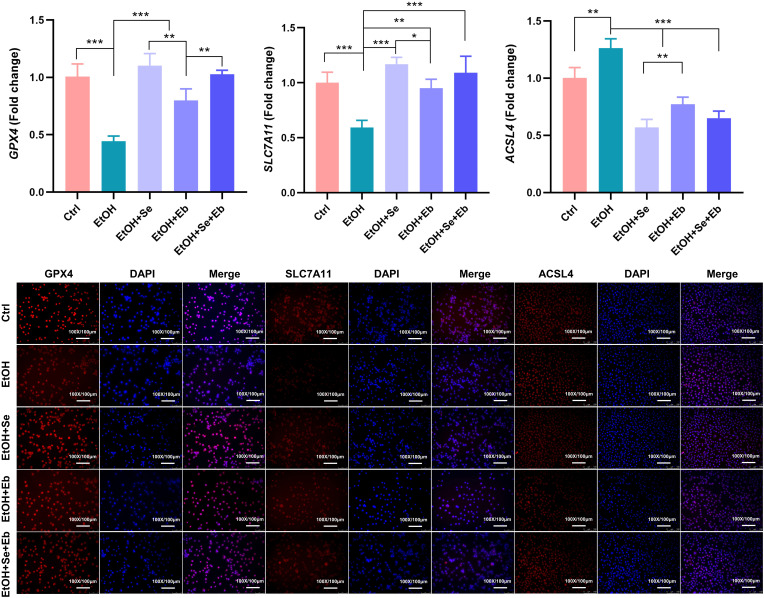
Effects of selenomethionine (Se) and/or ebselen (Eb) on the expression of ferroptosis-related genes of ethanol-induced hepatocyte damage. The mRNA expression level and representative immunofluorescence staining of GPX4, SLC7A11, and ACSL4 in NCTC clone 1469 after ethanol intoxication in the presence of Se and/or Eb treatments, scale bar = 100 μm. Data from each group were expressed as mean ± SD (n = 3). * means* P *< .05, ** means* P *< .01, and *** means* P *< .001 between indicated groups.

The changes in mRNA and protein levels of GPX4, SLC7A11, and ACSL4 genes, which are tightly correlated with the ferroptosis pathway, were detected in the cells of each treatment group (Figure 4). Compared with the control group, the levels of GPX4 and SLC7A11 were significantly reduced (all *P *< .001), whereas the level of ACSL4 was remarkably increased in the ethanol-treated group (*P *= .002), with all differences being statistically significant. However, the addition of Se and/or Eb pretreatment restored the altered expression of these genes compared to the ethanol-treated group. Selenium compound pretreatment significantly increased the levels of GPX4 and SLC7A11, while reducing the expression of ACSL4 (all *P *< .001). The Se and Se+Eb intervention groups showed more pronounced changes in gene expression levels. Immunofluorescence assays also demonstrated that ethanol exposure attenuated the expression of GPX4 and SLC7A11 while enhancing the level of ACSL4, further confirming the above findings. These results suggest that selenium compounds may also inhibit the process of ethanol-induced hepatocyte injury by regulating the ferroptosis pathway.

### Selenium and Ferrostatin-1 Attenuated Ethanol-induced Hepatocyte Damage In Vivo

**Figure 5.**
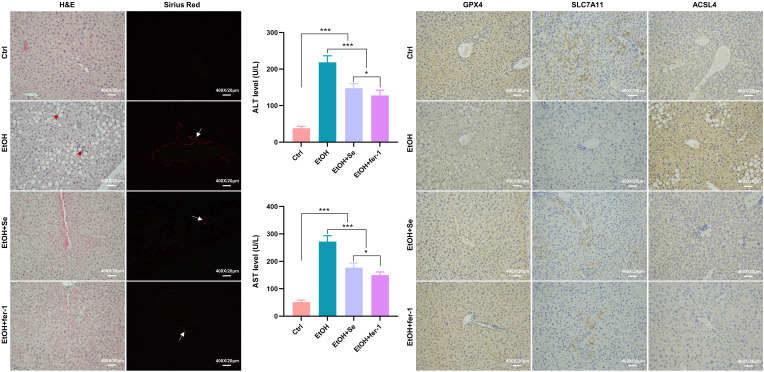
Selenomethionine and ferrostatin-1 prevented ethanol-induced liver injury in vivo. Representative hepatic H&E and Sirius Red staining of control mice, mice with ethanol, with or without selenomethionine or ferrostatin-1 (fer-1) administration, red arrows indicate inflammatory cell infiltration and vacuolation, white arrows indicate the distribution of collagenous fiber under polarized light, scale bar = 20 μm. Serum indices of ALT and AST of control, ethanol, with or without selenomethionine or fer-1 administration. The expressional change of hepatic GPX4, SLC7A11, ACSL4 in the liver tissue of control mice, and mice with ethanol, with or without selenomethionine or fer-1 administration. Data from each group were expressed as mean ± SD (n= 6). * means* P *< .05, ** means* P *< .01 and *** means* P *< .001 between indicated groups.

To further validate the role of selenium and fer-1 in an in vivo alcoholic hepatocyte injury model, we used the NIAAA approach to induce a chronic/binge drinking ALD model. Compared to control mice, the mice given ethanol exhibited blurred hepatocyte boundaries, disorganized hepatic cords, accompanied by inflammatory cell infiltration, vacuolation, and collagen formation. The hepatic histology evidently ameliorated after the intervention of selenomethionine or fer-1, with improvements observed in ballooning hepatocytes, focal inflammation, and collagen formation. Furthermore, serum ALT and AST levels were significantly higher in the ethanol group than in the control group, but they decreased after the intervention of selenomethionine or fer-1, with statistically significant differences (all *P *< .001). Moreover, the changes in GPX4, SLC7A11, and ACSL4 protein levels in liver tissues also reflected the role of the ferroptosis pathway in the mitigation of alcohol-induced hepatocyte injury by selenium (Figure 5).

## Discussion

ALD is a major global health problem. Alcohol is responsible for 50% of chronic liver disease deaths in Western countries and is the second leading cause of liver damage in China. According to statistics from the World Health Organization in 2018, excessive alcohol consumption leads to 3 million deaths per year, accounting for 5.3% of global deaths.^11^ Oxidative damage from alcohol and its metabolites is a key factor in promoting the development of ALD, and ALD causes selenium depletion in the body, while selenium deficiency exacerbates ALD, creating a positive feedback loop.^7^ Selenium, as an essential trace element for human health, is an important component of the body’s antioxidant defense system and immune functionality. It is known for its strong hepatoprotective bioactivity. Therefore, we analyzed the effects of selenium compounds in improving ALD and the potential mechanisms of cell death involved by establishing in vivo and in vitro models of ethanol-induced hepatocyte injury, with a view to providing an experimental basis and theoretical foundation for the use of selenium compounds in the treatment of liver diseases.

In this study, we first analyzed the effects of selenium compounds on alcohol-exposed NCTC clone 1469 cells. Our results showed that the intervention of selenium compounds significantly increased cell viability and improved lipid peroxidation levels in hepatocytes after ethanol injury, in which selenomethionine had a more pronounced effect compared to ebselen, which may be related to the differences in chemical structure and main biological effects. Currently, reported selenium compounds for anti-ALD injury are predominantly selenomethionine, such as selenium-enriched peanut protein and Bifidobacterium longum.^12-14^ In vivo animal experiments in this study also showed that treatment with selenomethionine significantly improved liver tissue damage and liver function index levels. All these results suggest that selenium-containing compounds have an ameliorating effect on ALD. Previous studies have shown that chronic drinkers have lower than normal selenium levels, possibly due to ethanol-induced oxidative damage inhibiting hepatic synthesis of selenoproteins such as albumin, which is positively correlated with serum selenium in ALD, and selenoprotein P, which plays a crucial part in the transport of serum selenium, and GPx, which responds to concentrations of elemental selenium.^7,15-16^ Related studies have also shown that moderate selenium supplementation alleviates oxidative stress induced by alcohol exposure, activates GPx activity, accelerates alcohol metabolism, regulates apoptosis, restores the function of antioxidant defense systems, and reduces hepatocyte vacuolation and liver fat mass.^17-18^ In the present study, we found that both apoptosis and ferroptosis, 2 types of cell death, were associated with the cytotoxic effects of alcohol metabolism. Intervention with selenium compounds improved apoptosis and partially restored the expression levels of altered ferroptosis pathway marker genes induced by ethanol. Studies have shown that apoptosis is closely associated with ferroptosis and that apoptosis can be converted to ferroptosis under certain conditions, with the latter promoting cellular sensitivity to apoptosis.^19^ A better understanding of the molecular mechanisms of cell death during alcohol injury to hepatocytes is essential for controlling disease progression.

Several studies have shown that ferroptosis is an important mechanism in the development of alcohol-induced liver disease. In this study, we analyzed the changes in molecules related to the ferroptosis pathway following the intervention of selenium compounds and ferroptosis inhibitors. The results demonstrated that the improvement of ALD by selenium compounds is regulated by the ferroptosis pathway. GPX4, SLC7A11, and ACSL4 are all crucial regulatory proteins of ferroptosis, and the decreased levels of GPX4 and SLC7A11 are regarded as markers of ferroptosis occurrence,^20^ modulating the expression of these proteins can promote or inhibit ferroptosis. It was shown that cellular sensitivity to ferroptosis was mainly regulated by GPX4 and ROS. Cells with decreased GPX4 expression were more susceptible to ferroptosis, while upregulated GPX4 expression confers tolerance to ferroptosis, and GSH could convert toxic lipid peroxides to non-toxic lipid alcohols under the catalytic action of GPX4.^21^ SLC7A11 is the light chain subunit of the cystine/glutamate reverse transporter (System Xc-), which constitutes an important cellular antioxidant system involved in GSH synthesis. The downregulation of SLC7A11 inhibits System Xc- uptake of cystine, leading to reduced activity of cystine-dependent GPXs, diminished cellular antioxidant capacity, and increased lipid ROS content, ultimately resulting in cellular ferroptosis.^22^ ACSL4 is the key enzyme responsible for the synthesis of polyunsaturated fatty acids in the ferroptosis pathway. These fatty acids contain unstable carbon-carbon double bonds that are susceptible to lipid peroxidation, which is necessary for the occurrence of ferroptosis.^23^ Our results suggest that ferroptosis plays an important role in the progression of ALD. Liu et al also demonstrated that ferroptosis inhibitors significantly attenuated alcohol-induced hepatocyte death and markedly improved alcoholic liver injury in mice.^8^ Therefore, blocking cellular ferroptosis may offer a potential therapeutic strategy to protect the liver from damage.

In summary, our data suggest that ferroptosis plays an important role in ethanol-induced hepatocyte injury, and selenium compounds or ferroptosis inhibitors can effectively alleviate ALD. Selenium compounds exert a protective effect on the liver by regulating apoptosis and ferroptosis through antioxidant effects. However, the specific molecular mechanism underlying the action of selenium is still to be elucidated. These studies will facilitate the further development of ALD treatment.
